# Role of cobalt cations in short range antiferromagnetic Co_3_O_4_ nanoparticles: a thermal treatment approach to affecting phonon and magnetic properties

**DOI:** 10.1038/s41598-017-18563-9

**Published:** 2018-01-10

**Authors:** Swati R. Gawali, Ashish Chhaganlal Gandhi, Shrikrushna Shivaji Gaikwad, Jayashree Pant, Ting-Shan Chan, Chia-Liang Cheng, Yuan-Ron Ma, Sheng Yun Wu

**Affiliations:** 1 Department of Physics, CES’s Dr. A. B. Telang Sr. College, Savitribai Phule Pune University, Pune, 411007 India; 2 Department of Physics, National Dong Hwa University, Hualien, 97401 Taiwan; 3 Department of Physics, Abasaheb Garware College, Savitribai Phule Pune University, Pune, 411007 India; 4 National Synchrotron Radiation Research Center, Hsinchu, 30076 Taiwan

## Abstract

We report the phonon and magnetic properties of various well-stabilized Co_3_O_4_ nanoparticles. The net valence in cobalt (II)/(III) cation can be obtained by subtracting the Co^2+^ ions in tetrahedral interstices and Co^3+^ ions in the octahedral interstices, respectively, which will possess spatial inhomogeneity of its magnetic moment via Co^2+^ in tetrahedra and Co^3+^ in octahedral configurations in the normal spinel structure. Furthermore, the distribution of Co^2+^/Co^3+^ governed by various external (magnetic field and temperature) and internal (particle size and slightly distorted CoO_6_ octahedra) sources, have led to phenomena such as a large redshift of phonon-phonon interaction and short-range magnetic correlation in the inverse spinel structure. The outcome of our study is important in terms of the future development of magnetic semiconductor spintronic devices of Co_3_O_4_.

## Introduction

Nanomaterials usually exhibit a number of unique and enhanced properties, which may strongly differ from those observed in their bulk counterpart. In the last decade, research on the effects of the finite size on the structural, optical and magnetic properties of Co_3_O_4_ have attracted enormous attention because of its wide range of important potential applications as a gas sensor^1^, data storage^2^, magnetic semiconductors^3^, electrochemical devices^4^, heterogeneous catalysts^5^, anode materials in Li-ion rechargeable batteries^6^, solid-state sensors^3^, solar energy absorbers, pigments^7–9^ etc. The functioning of all these devices is strongly influenced by the different sample synthesis methods^3^, point defects (such as cation or anion vacancies)^10^, their morphology (such as nanobelts, nanorods, nanospheres, nanocubes, nanowires and nanoflowers) and nanosized effect^11,12^. Recently, Casas-Cabanas *et al*.^7^ carried out a crystal structural investigation of Co_3_O_4_ nanoparticles (NPs) with size varrying from 32 nm to a few micrometers. In their comprehensive analysis, they discovered that small size particles having high cobalt vacancies exhibit a strong variation in the intensity ratio of (111)/(220) XRD diffraction peaks. Similar high intense (111) diffraction peak from Co_3_O_4_ have also been reported by Furlanetto^13^ and Cao *et al*.^14^. Co_3_O_4_ has a simple spinel structure ($Fd\bar{3}m$) having oxygen ions (32e Wyckoff sites) slightly displaced from the ideal (1/4, 1/4, 1/4) position with cations Co^2+^(8a Wyckoff sites) and Co^3+^(16d Wyckoff sites) distributed among tetrahedral and octahedral sites, respectively. It also exhibits *p*-type semiconducting properties^15^ because of cationic (cobalt) vacancies resulting in an oxygen rich spinel. However, unlike mono-oxides, the choice of the cationic vacancy location in a spinel structure is much more complex as it could be either on Co^2+^ sites, Co^3+^ sites, or both. Angelov *et al*.^16^ deduced from electron paramagnetic resonance (EPR) analysis that cobalt vacancies in oxygen-rich Co_3−x_O_4_ powder resides on octahedral sites. Furthermore, most of the above applications of Co_3_O_4_ are based on the use of nanomaterials with magnetic properties that often differ considerably from those of their bulk counterpart. Bulk Co_3_O_4_ is an antiferromagnetic (AFM)^17^, having a Néel temperature *T*_*N*_ between 30 K^18,19^ and 40 K^17^. Resnick *et al*.^20^ reported superparamagnetic (SPM) temperature *T*_*B*_ ~ 5.4 K and reduced *T*_*N*_ = 15 ± 2 K from 4.34 nm Co_3_O_4_ nanoparticles. The observed SPM behavior and reduced *T*_*N*_ was ascribed to uncompensated spins, which are attributed to structural inhomogeneities, defects, and finite size effects.

Here, it is important to note that magnetic moments in Co_3_O_4_ arise solely because of spins of Co^2+^ ions having a small contribution from spin-orbit coupling^17^. On the other hand, the Co^3+^ ions at the octahedral sites are diamagnetic as a consequence of the splitting of 3d levels by the octahedral crystal field and complete filling of *t*_*2g*_ levels due to which vacancies at octahedral sites do not contribute to net magnetization. Therefore, apart from XRD and Raman measurements, a rigorous magnetic study of Co_3_O_4_ nanoparticles having cobalt vacancies can help us in locating their sites in the spinel structure. In this study, details of the synthesis, as well as the structural and magnetic properties of various sizes of Co_3_O_4_ nanoparticles are presented. This work aims to investigate the influence of cobalt cations and the finite size effect on the induction of short-range magnetic correlation and Néel temperature.

## Results

### Morphological and stoichiometric analysis

**Figure 1:**
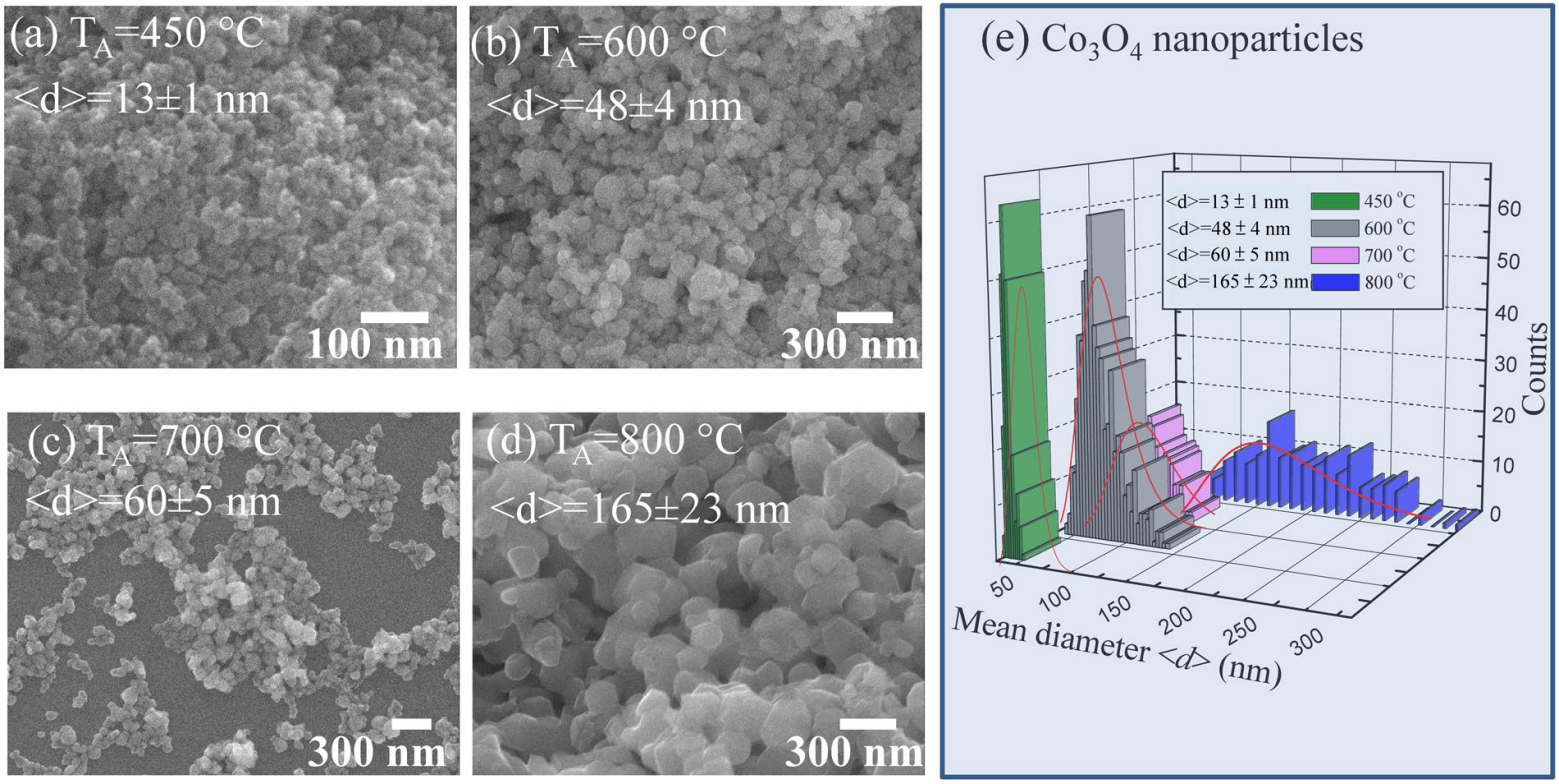
(**a**)–(**d**) A portion of SEM images of 450 °C to 800 °C annealed samples representing size distribution and morphology. (**e**) Histogram of diameter distribution of Co_3_O_4_ NPs of various samples calculated from the SEM images, where the solid-line represents log-normal distribution function fit.

**Figure 2:**
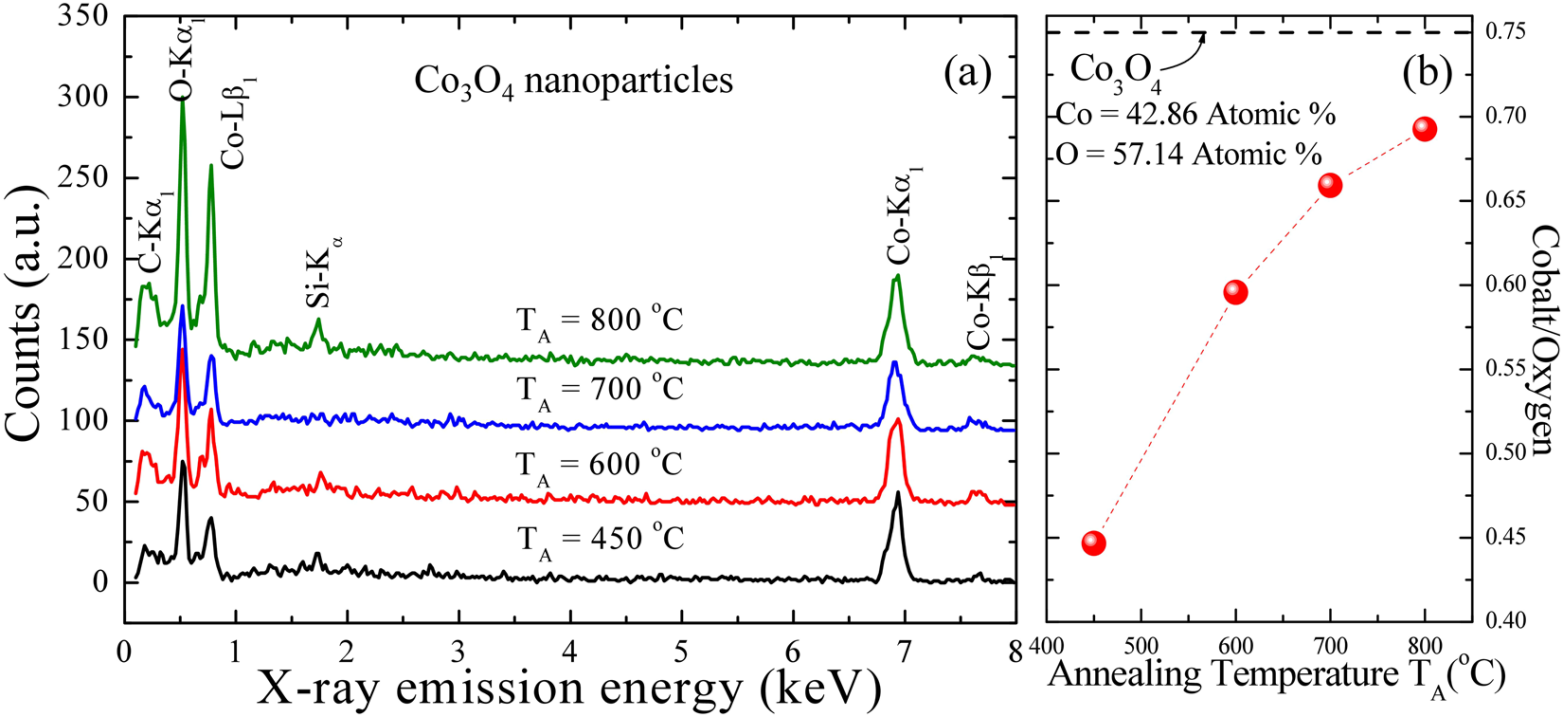
(**a**) Plot of EDS spectra for Co_3_O_4_ NPs, which are shifted vertically for clear visibility. (**b**) The variation in the ratio of cobalt/oxygen with respect to the crystalline size of NPs.

The well dispersed and pseudo-spherical nanoparticles of Co_3_O_4_ are visible from a portion of SEM images after annealing at 450, 600, and 700 °C, as shown in Fig. 1(a)–(c). The interconnecting nanoparticles were stuck together in clusters because of electrostatic effects as well as an artifact of the drying of the aqueous suspension, revealing a nanoparticle agglomeration behavior. The clustering makes an evaluation of the size distribution possible. A similar behavior of nanoparticle agglomeration has also been reported from NiO^21^, ZnO^22^ nanoparticles, and NiO nanowalls^23^. However, the samples annealed at 800 °C (Fig. 1(d)) formed sinter necks between them, showing a sintering behavior. To estimate the mean size of nanoparticles, the distribution of particle diameter can be calculated from the SEM images and described by using log-normal distribution function, as shown in Fig. 1(e). The log-normal distribution function is defined as follows:$f(d)=\frac{1}{\sqrt{2\pi }d\sigma }\,exp[-\frac{{(lnd-ln < d > )}^{2}}{2{\sigma }^{2}}]$, where <*d*> is the mean diameter and σ is the standard deviation of the function. The mean diameter and standard deviation of Co_3_O_4_ nanoparticles annealed at 450, 600, 700, and 800 °C were obtained after fitting the log-normal function, that is <*d*> = 13 ± 1, 48 ± 4, 60 ± 5, 165 ± 21 nm and *σ* = 0.23 ± 0.11, 0.30 ± 0.09, 0.27 ± 0.09, 0.41 ± 0.16 nm, respectively. The value of *σ* is below 0.3 nm for nanoparticles annealed at 450, 600, and 700 °C indicating the distributions of nanoparticles is confined within a narrow range^24^, whereas the distribution of nanoparticles annealed at 800 °C is confined within the intermediate range^25^. Energy-dispersive spectroscopy (EDS) technique is used to detect the chemical purity and estimate the atomic percentage in a given sample^23,24^. The EDS spectra of various annealing temperatures plotted in Fig. 2(a) are associated with a series of elemental cobalt and oxygen, that can be assigned to Co*-*L*β*_1_, Co*-*K*α*_1_, Co*-*K*β*_1_, and O*-*K*α*_1_. The small, intense peaks of C*-*K*α*_1_ and Si-Kα are originated from the contribution of carbon film on the Cu-grid and the silicon substrate as a consequence of mounting the sample^21^.The estimated atomic percentage ratio of Co/O obtained from EDS spectra reveals an increasing behaviour with the annealing temperature and approaches to a value of 0.692 after annealing at 800 °C. The value is close to the theoretical stoichiometric value of 0.75^10^ for Co_3_O_4_ as shown in Fig. 2(b). Significantly, a small Co/O atomic percentage ratio of 0.45 obtained in the sample annealed at 450 °C, has attracted more interest, indicating a huge deficiency of Co in the sample and ~0.6 times that of the bulk value. The effect of annealing in an ambient atmosphere resulted in an increase of the Co/O ratio, which approached to the value of bulk stoichiometry after annealing at 800 °C. George *et al*.^10^ also reported a similar effect of annealing on the stoichiometry of nano-crystalline Co_3_O_4_ ultra-fine fibers synthesized by sol-gel electrospinning. In their analysis, they observed the effect of the annealing result in an increase of the crystallinity, grain size (<*d*> = 25.3 nm at 400 °C and <*d*> = 42.9 nm at 500 °C) of Co_3_O_4_ nano-fibers. However, there are also reports in which either a slightly increased^26^ or almost similar^11,27^ value of Co/O atomic percentage ratio as that of the theoretical value has been reported for various sized Co_3_O_4_ nanoparticles synthesized by different chemical and physical techniques. This synthesis method plays an important role in defining the stoichiometry and crystallinity of nanomaterials. In this set of samples, the observed cobalt vacancies could be either at octahedral (Co^3+^), tetrahedral (Co^2+^) sites or at both the sites of Co_3_O_4_, which will be discussed later in the text.

### X-ray diffraction and analysis

**Figure 3:**
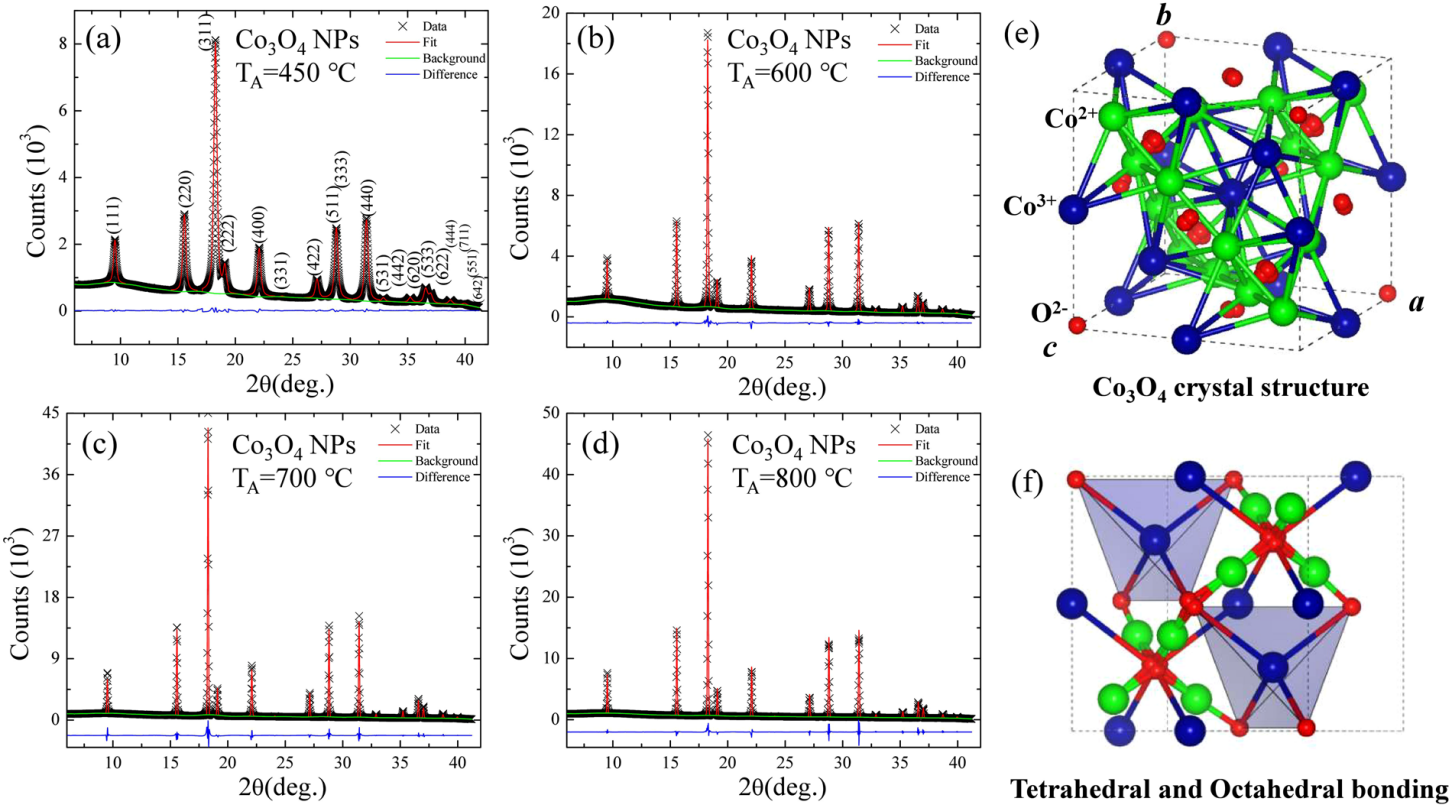
(**a**)–(**d**) Observed x-ray patterns (black crosses) of Co_3_O_4_ NPs synthesized at various annealing temperatures T_A_ = 450, 600, 700, and 800 °C, respectively. The solid red lines are the Rietveld refinement fit to x-ray patterns. The difference (blue curve) between the observed and the fitted patterns is plotted at the bottom of figure. (**e**) Crystal structure of Co_3_O_4_ NPs. (**f**) Plot of Co^2+^ ions in tetrahedral interstices and Co^3+^ ions in the octahedral interstices of the cubic close-packed lattice of oxide anions.

**Figure 4:**
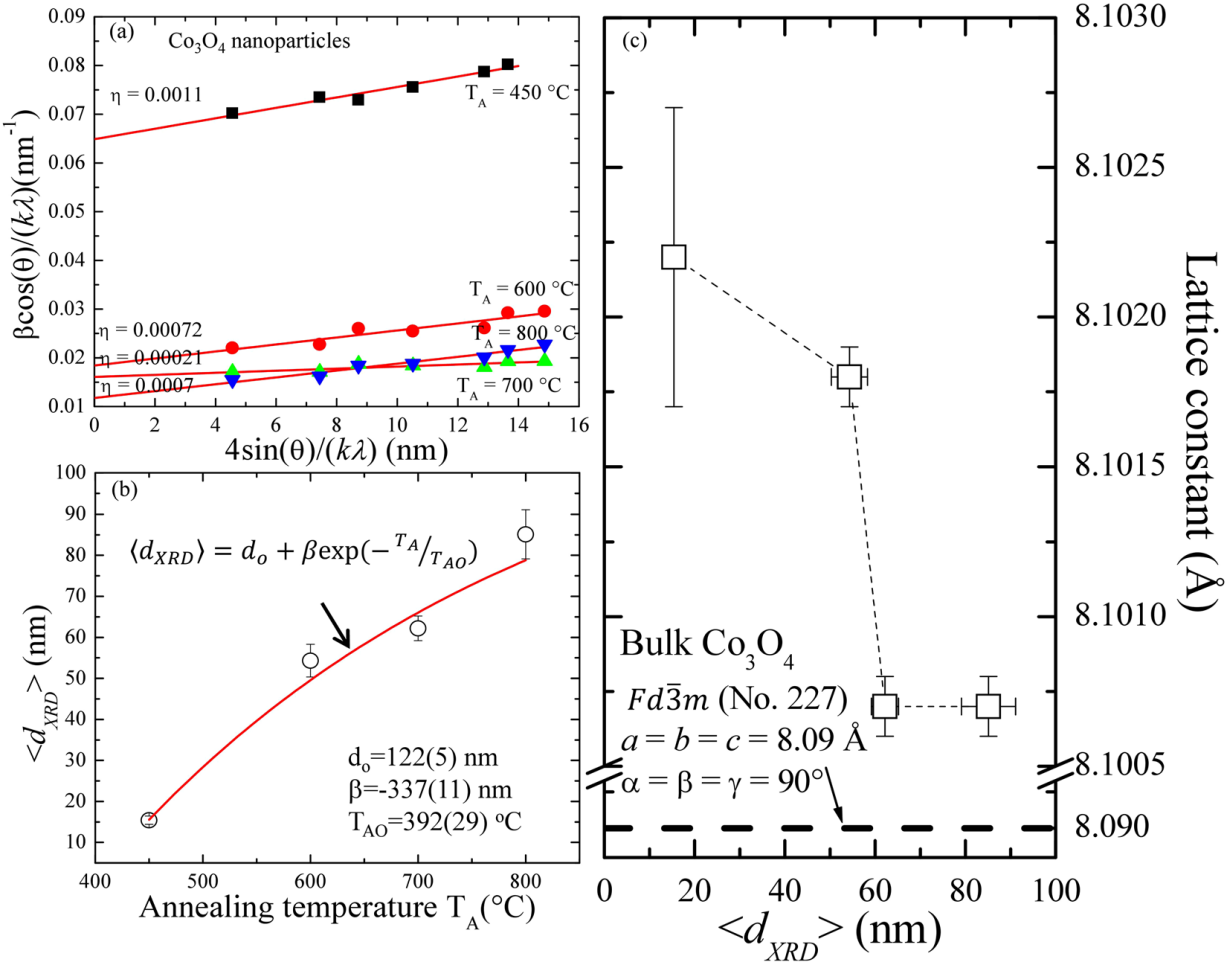
(**a**) Linear fit obtained using the Williamson−Hall correlation for each Co_3_O_4_ NPs, where the slope of the graph gives the mean strain and the intercept of the line with the y axis gives the inverse particle size. (**b**) Annealing temperature T_A_ dependence of the mean nanoparticle diameter $< {d}_{XRD} >$, where the solid line shows the fit to the exponential function and the fitted values are indicated. (**c**) Plot of lattice constant with respect to nanoparticle size obtained from the Williamson–Hall plot showing lattice expansion with decreasing particle size.

**Figure 5:**
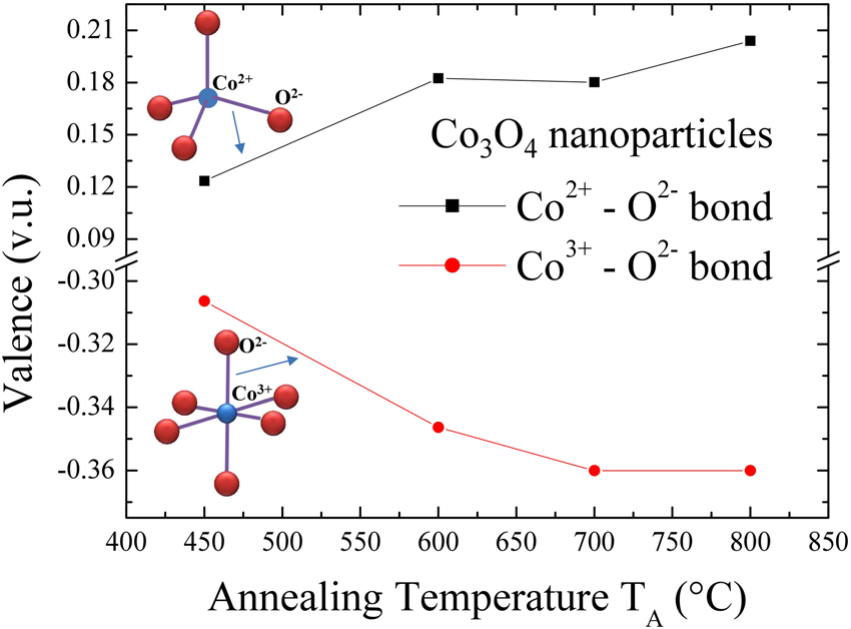
Plot of annealing temperatures dependence of the net valence of Co^2+^(0 and Co^3+^(0), respectively.

The detailed crystalline structure and strain in various sized Co_3_O_4_ nanoparticles can be investigated by using a high-energy synchrotron x-ray diffraction technique. For the purpose of structural analysis, the Rietveld refinement of x-ray patterns of different Co_3_O_4_ samples was carried out by using a GSAS software package. Figure 3 (a)–(d) display the x-ray diffraction patterns taken at various annealing temperatures, where the red, green, and blue curves indicate the fitted pattern, the background, and the difference between the observed and fitted pattern, respectively. As we know, Co_3_O_4_ adopts the normal spinel structure shown in Fig. 3(e) with Co^2+^ ions in tetrahedral interstices and Co^3+^ ions in the octahedral interstices of the cubic close-packed lattice of oxide anions, as plotted into Fig. 3(f). The obtained refined lattice parameters indicate that the nanoparticles are crystallized as a cubic spinel cobalt oxide (Co_3_O_4_) with a space group of $Fd\bar{3}m$ (No. 227) without any trace of additional impurity. The fitted parameters are summarized in the supplementary information of Table S1. Significant broader diffraction peaks are clearly visible through naked eyes from the x-ray pattern of the sample annealed at 450 °C, in agreement with the nanometric nature of the sample as can be seen in Figure 3(a) ^7^. With an increase of annealing temperature, diffraction peaks and the full width at half-maximum (*fwhm*) become sharper and broaden, as a result of an increase in crystalline size and the loss of internal microstrains^7^. The observed broadening of the diffraction peak reveals a short-range crystalline behavior that can be fitted by the Gaussian function^28^. The value of *fwhm* of the most intense diffraction peak [311] tabulated in the supplementary information of Table S1 showed a rapid increase from 0.078 ± 0.003 to 0.308 ± 0.002 as the grain size decreased from the 85 ± 6 nm to 15 ± 1 nm, signaling the finite size effect or the combined effect of size and strain. The grain size ${d}_{XRD}$ and the strain (*η*) of various sized Co_3_O_4_ nanoparticles were estimated by using Williamson-Hall (W-H) plot^29^ and the well-known Scherrer formula^30^ $\beta ={\beta }_{size}+{\beta }_{strain}=\frac{1}{cos\theta }(\frac{k\lambda }{ < {d}_{XRD} > }+4\eta sin\theta )$, where *β* is the *fwhm* of the diffraction peak, *k* is the Scherrer constant ( = 0.94) for spherical nanoparticles, *θ* is the diffraction angle, *λ* is the incident x-ray wavelength, and *η* is the strain parameter, respectively. Figure 4(a) shows the plot of βcosθ/kλ versus 4sinθ/kλ for all Co_3_O_4_ samples. The intercept of the linear fit to the data point in the W-H plot gives the value of grain size ${d}_{XRD}$, whereas the slope presents the value of strain. The estimated value of strain is below 0.0011, indicating that nanoparticles are free from any strain and therefore the broadening of diffraction peaks is exclusively because of the finite size effect. The value of strain is close to the estimated value of 0.00095 from the W-H plot reported from 15 nm size Co_3_O_4_ nanoparticles synthesis by using a microwave technique^12^. Rao *et al*.^31^ reported a value of η = 0.0070 from ~13 nm size Co_3_O_4_ nanoparticles synthesized by a urea-based combustion method which is ~7 times higher than the observed value. The observed grain size after extrapolating the linear fit in the W-H plot of Co_3_O_4_ nanoparticles annealed at 450, 600, 700, and 800 °C were 15 ± 1, 54 ± 4, 62 ± 3, and 85 ± 6 nm, respectively. The observed grain size of 15 ± 1, 54 ± 4, and 62 ± 3 nm (from W-H plot) and mean nanoparticle diameter of 13 ± 1, 48 ± 4, and 60 ± 5 nm (from the portion of SEM images) of various annealed Co_3_O_4_ nanoparticles at T_A_ = 450, 600 and 700 °C, respectively, are in excellent agreement with each other, revealing the formation of nanoparticles with a single domain. However, the grain size of Co_3_O_4_ nanoparticles annealed at T_A_ = 800 °C, 85 ± 6 nm obtained from x-ray diffraction differs significantly from the result of SEM (<d> = 165 ± 23 nm). The discrepancy of obtained particle size is due to the agglomeration behavior of nanoparticles at higher annealing temperatures, which resulted in the formation of multiple domains within each nanoparticle^21–23^. The corresponding particle size ${d}_{XRD}$ of the Co_3_O_4_ nanoparticles versus the annealing temperatures are shown in Fig. 4(b). As seen in Fig. 4(b), the estimated values of ${d}_{XRD}$ versus the annealing temperature T_A_ are plotted, revealing an increase with the increase in the particle size. The *red solid curve* indicates the fit of the data to the theoretical curve for an exponential function, $\langle {d}_{XRD}\rangle ={d}_{o}+\beta \exp (-{T}_{A}/{T}_{AO})$, where d_o_ = 122(5) nm, β = −337(11) nm, and T_AO_ = 392(29) °C represents the initial constant and the fitted parameters, respectively. A slight lattice expansion of 0.15% (percent deviation) was observed from the T_A_ = 450 °C sample and as the annealing temperature T_A_ increased further, it approaches to the bulk value of ~8.09 Å^19^ as shown in Fig. 4(c). A similar lattice expansion has been reported previously from Co_3_O_4_ nanoparticles synthesized by chemical means^7,32^. Moreover, many metal oxides show lattice expansion with a reduction of particle size which could be either because of the finite size effect, cation/anion vacancies, lattice stress *etc*. However, in general, Co_3_O_4_ nanoparticles synthesized by using different methods exhibit the common trend of lattice expansion with an increase of particle size^33–35^. For charge neutrality, some of the Co^3+^ vacancies could be substituted by Co^2+^ ions, which resulted in the contraction of the Co^2+^–O^2−^ bond length. Therefore, further investigations of net valences are expected in the next step. This can be calculated quantitatively using the bond valence method developed by Brown and O’Keefe^36^. The general principles of the bond valence method can be summarized as ${v}_{jk}=exp[({R}_{jk}-{d}_{jk})/b]$, where ${v}_{jk}$ is the bond valence associated with a bond of length ${d}_{jk}$ between the neighboring atoms *j* and *k*, the bond valence parameter ${R}_{jk}$ is the distance corresponding to a bond valence *v* = 1.0 v.u. and ${V}_{j}$ is the sum valence of $\sum _{k}{v}_{jk}$. The universal constant b is equal to 0.37 Å. The net valence in the Co^2+^-O^2−^ bond and the Co^3+^-O^2−^ bond can be obtained by subtracting the Co^2+^ ions in tetrahedral interstices and the Co^3+^ ions in the octahedral interstices, respectively. The net valences of Co^2+^ and Co^3+^ at various annealing temperatures are summarized in the supplementary information of Table S2 ^.^ One of the important differences in our study from earlier reported crystal structures (bulk Co_3_O_4_^19^) lies in the typical bond distances of Co^2+^-O^2−^ and Co^3+^-O^2−^. The Co^2+^-O^2−^ bond lengths (obtained from the Rietveld refined structure) vary from 1.917 Å to 1.91 Å in the present study as compared with 1.929 Å as reported earlier^19^_._ In additione typical bond distance of Co^3+^-O^2−^ in our case ranges from 1.935 to 1.938 Å as compared to that reported earlier value of 1.916 Å in slightly distorted CoO_6_ octahedra. It may also be possible that some of the Co sites will have Co^3+^ instead of Co^2+^ which would further give rise to the decreasing of net valence Co^3+^(0) and the increasing of net valence Co^2+^(0) with increasing annealing temperature, as can be seen in Fig. 5. This might result in the shortening of the Co^2+^-O^2−^ bond lengths accompanied by an increase in Co^3+^-O^2−^ bond lengths at least for that oxygen which is connected to both Co^2+^ and Co^3+^, which will possess spatial inhomogeneity of its magnetic moment via Co^2+^ in tetrahedra and Co^3+^ in octahedral configurations in the normal spinel structure. However, the distribution of Co^2+^/Co^3+^ governed by various external sources like magnetic field, high pressure, temperature etc., have led to phenomena like diffusive charge transfer, a very large redshift of phonon-phonon interaction in the inverse spinel structure, as will be discussed in the Raman and magnetization analysis session.

### Phonon Excitation in Co_3_O_4_ nanoparticles

**Figure 6:**
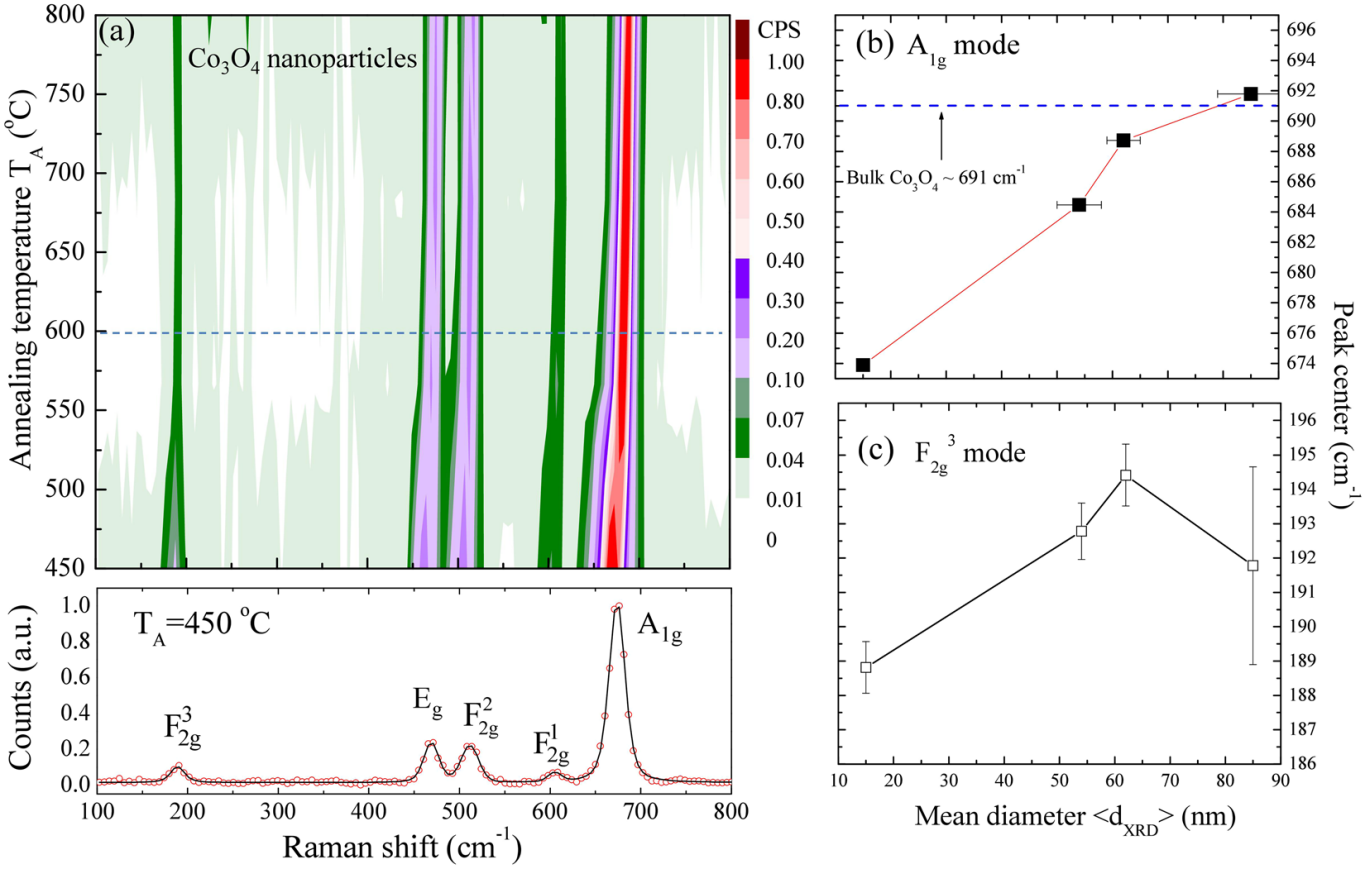
(**a**) Two-dimensional map of the intensity and T_A_ dependence of Raman patterns for Co_3_O_4_ NPs taken at room temperature. A selected Raman pattern taken at T_A_ = 450 °C is shown at the bottom, revealing a series of phonon modes excitations. Crystalline size dependency of (**b**) A_1g_ phonon peak center and (**c**) ${{\rm{F}}}_{2{\rm{g}}}^{3}\,$mode, respectively.

Confocal Raman spectroscopy was utilized to study the size dependence of related phonon coupling of the cobalt cation of Co_3_O_4_ nanoparticles due to their high spatial resolution and sensitivity for probing atomic vibration. Figure 6(a) displays the series of Raman spectra for annealing temperatures ranging from 450 °C to 800 °C (bottom to top). Five main phonon excitations were observed at T_A_ = 450 °C, as shown at the bottom of Fig. 6(a), which are in good agreement with the values predicted by the group theory and the related report of the Co_3_O_4_ nanoparticles^37,38^, consisting of ${{\rm{F}}}_{2{\rm{g}}}^{3}$ + E_g_ + ${{\rm{F}}}_{2{\rm{g}}}^{2}$ + ${{\rm{F}}}_{2{\rm{g}}}^{1}$ + A_1g_^39–42^. The band at 674 cm^−1^ is viewed as the symmetric Co-O stretching vibration of the octahedral (CoO_6_) group, which is assigned to the A_1g_ species in the ${O}_{h}^{7}$ spectroscopic symmetry. The Raman bands with medium intensity located at 469 and 511.5 cm^−1^ have E_g_ and ${{\rm{F}}}_{2{\rm{g}}}^{2}$ symmetry, respectively, whereas the weak band located at 605 cm^−1^ has the ${{\rm{F}}}_{2{\rm{g}}}^{1}\,$symmetry. The band at 188.8 cm^−1^ is attributed to the characteristics of the tetrahedral sites (CoO_4_), which are attributed to the ${{\rm{F}}}_{2{\rm{g}}}^{3}$ symmetry. This result further confirms the formation of the Co_3_O_4_ nanocrystals. The growth temperature T_A_ dependence of the phonon peak positions and intensities obtained from two-dimensional Raman images of Co_3_O_4_ nanoparticles are shown at the top of Fig. 6(a), where different colors present the peak intensity of the Raman patterns. As we can see from Fig. 6(a) below T_A_~ 600 °C, a significant shoulder is observed on the higher frequency of A_1g_ mode near the lower T_A_ regime of the Co_3_O_4_ nanoparticles. The quantitative analysis of anomalous behavior was carried out by peak profile fitting using the Voigt distribution function covering the whole regime. The detailed T_A_ dependences of the peak position and full widths at half maximum (*fwhm*) are summarized in the supplementary information of Table S3. Figure 6(b)–(c) presents the evolution of the peak center of the two selected A_1g_ and ${{\rm{F}}}_{2{\rm{g}}}^{3}$ modes related to the vibrations of the octahedral position occupied by the Co^3+^ cation and the tetrahedral position occupied by Co^2+^ cation for various T_A_, respectively. As the particle size ${d}_{XRD}$ is reduced, the peak center of the A_1g_ mode rapidly shifts to a lower wave number by about 17 cm^−1^ (in comparison with the bulk value of 691 cm^−1^) while simultaneously the ${{\rm{F}}}_{2{\rm{g}}}^{3}$ mode down shifts about 3 cm^−1^, revealing the finite size effect. Peak shift and asymmetrical broadening of the Raman line shape may lead to the important information related to the present Co_3_O_4_ nanoparticles under investigation. Tensile stress causes the Raman band to red shift. Lattice disorder and low dimensional crystals lead to the asymmetrical broadening and the downshifting of the A_1g_ mode. But another factor that results in an asymmetrical broadening and a shoulder on the higher frequency is the thermal heating process, which results in an increase of the lattice parameter near the lower T_A_ regime of Co_3_O_4_ nanoparticles, as can be found in the supplementary information of Table S1. This observation cannot be explained in terms of a change in stoichiometry with the decrease of particle size. Thus, the observed Raman shift and line-broadening is attributed to a size-dependent effect on the nanoparticles which is in good agreement with the previous reports^43^.

### Isothermal Magnetization Measurement

**Figure 7:**
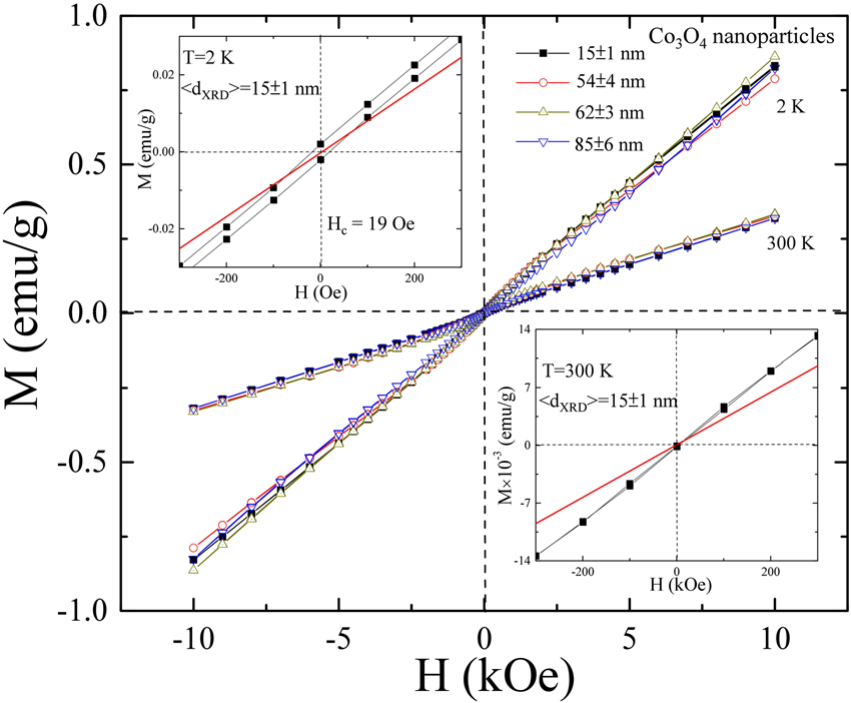
Isothermal hysteresis M(*H*) loop measured at 2 and 300 K for all Co_3_O_4_ NPs. In the inset of figure is the zero-field region M(*H*) loop for 15 ± 1 nm NPs measured at 2 K (1^st^ quadrant) and 300 K (4^th^ quadrant).

Isothermal magnetization hysteresis loop *M*(*H*_*a*_) measurement of Co_3_O_4_ nanoparticles was taken at temperatures 2 K and 300 K over a magnetic field of ± 10 kOe, as shown in Fig. 7. A coercivity *H*_*C*_ = 19 Oe is observed for 15 ± 1 nm NPs at 2 K (shown in 1^st^ quadrant of the figure), thus exhibiting weak FM behavior of Co_3_O_4_ NPs and paramagnetic (PM) behavior at room temperature (shown in 4^th^ quadrant figure). For bulk AFM material, below Néel temperature *T*_*N*_, magnetization is expected to vary linearly with an applied field with zero coercivity (*H*_C_) and remanence (*M*_*r*_)^19^. The observed zero value of *H*_*C*_, *M*_*r*_ and non-saturating behavior of *M*(*H*_*a*_) loop from Co_3_O_4_ nanoparticles measured at 2 K (below *T*_*N*_) indicates the pure AFM behavior. Magnetic moments in Co_3_O_4_ arise solely because of spins of Co^2+^ ions having a small contribution from spin-orbit coupling^17^. On the other hand, the Co^3+^ ions at the octahedral sites are diamagnetic as a consequence of the splitting of *3d* levels by the octahedral crystal field and complete filling of *t*_*2g*_ levels due to which vacancies at the octahedral sites do not contribute to net magnetization. The observed linear increasing behavior of *M*(*H*_*a*_) loop above and below *T*_*N*_ is in good agreement with the previously reported mesoporous Co_3_O_4_ nanostructures^42^. However, in general, AFM metal oxide nanoparticles exhibit spin-glass and/or weak-ferromagnetic (FM) like behavior arising from the uncompensated surface spins^27,44,45^. The randomly oriented, uncompensated surface spins form the short-range ordered clusters of the spins which behave like weak-FM. Recently, Valan *et al*.^26^ reported the observation of soft and weak-FM behavior from 45 and 29 nm Co_3_O_4_ nanoparticles synthesized by using microwave combustion method, which was ascribed to uncompensated surface spins and/or finite size effect. From the magnetic point of view, such nanoparticles behave like a core-shell having an uncompensated AFM core and a short-range ordered weak-FM shell. The intercoupling between the spins of AFM and FM at the interface results in the formation of a unidirectional anisotropy energy barrier, which gives rise to a hysteresis loop shift called an exchange bias phenomenon. The exchange bias phenomenon could be conventional (CEB)^46^ or spontaneous exchange bias (SEB)^47^. Zeng *et al*.^35^ reported the observation of a CEB field of −600 Oe from 25 nm Co_3_O_4_ nanoparticles using a cooling field of 50 kOe arising from the intercoupling between a spin-glass-like shell and an uncompensated AFM core. Wang *et al*.^42^ also reported the observation of a CEB field of −530 Oe at 5 K from nanoporous Co_3_O_4_ rods (~30 nm nanocrystals) using a cooling field of 10 kOe arising from the intercoupling between the nanocrystals within the nanorods. During the last decades, several reports on Co_3_O_4_ nanoparticles have claimed the observation of the CEB effect with a maximum loop shift of −800 Oe using a cooling field of 70 kOe^19,48–51^. Similarly, a CEB effect due to the intercoupling between spins of the spin-glass-like or weak-FM shell and uncompensated AFM core has also been reported from nanoparticles of various AFM metal oxides such as NiO^52^, CoO^53^, MnO^54^, Cr_2_O_3_^55^ etc. However, the observation of such a high exchange bias field seems to be overestimated, which could be due to the pre-magnetization of surface spins in an external cooling field. Since, the value of CEB is dependent on several factors such as interfacial roughness, the complex spin structure, uncompensated spins at the interface, and particularly thickness that is on the long-range ordering of FM and AFM components as observed from several core-shell FM/AFM nanoparticles. In the last few years, the SEB effect has been observed in various systems from bulk, FM/AFM core/shell, and nanocomposite to a pure AFM nanostructure^56^. In our previous magnetic study of a NiO nanoparticles system^57^, we also observed that the SEB field decreases with the increase of particle size. Therefore, for a proper understanding of the hysteresis loop shift in Co_3_O_4_ nanoparticles, a comprehensive magnetic study is needed. Furthermore, above the Néel temperature, the thermal energy overcomes the AFM ordering, and the material behaves paramagnetically. The *M*(*H*_*a*_) loop measured at 300 K shows paramagnetic like behavior with a zero value of *H*_*C*_, *M*_*r*_ with linear increasing and non-saturating behavior of the magnetization with an applied magnetic field.

### Temperature dependence of the magnetization measurement

**Figure 8:**
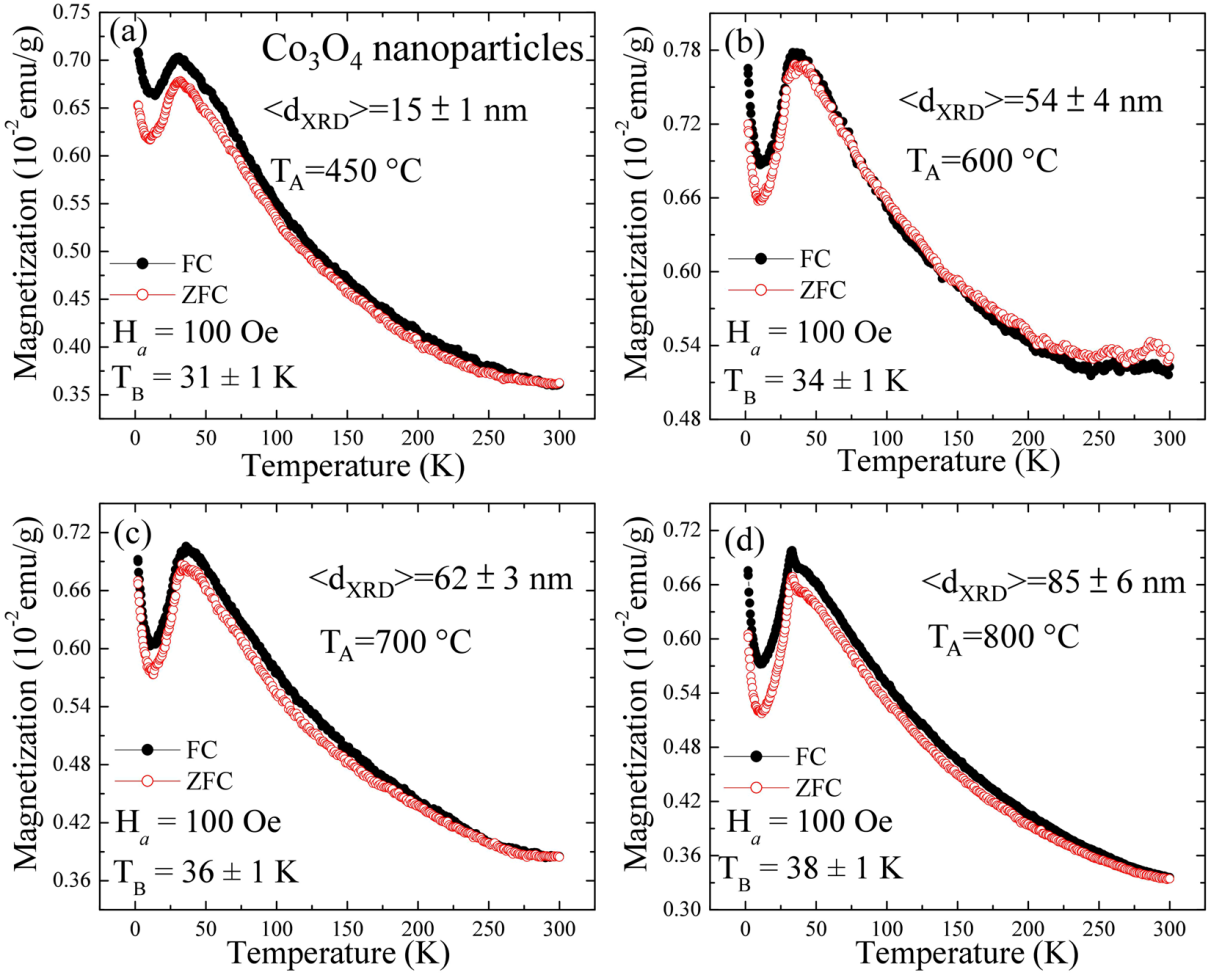
(**a**)–(**d**) Temperature dependent magnetization M(*T*) measured by using ZFC-FC process with 100 Oe applied field for 15 ± 1, 54 ± 4, 62 ± 3 and 85 ± 6 nm Co_3_O_4_ NPs.

The magnetic susceptibility of bulk AFM material increases with a measuring temperature below the *T*_*N*_ while above, it exhibits paramagnetic behavior^19^. To determine the temperature dependence of the magnetic moment of Co_3_O_4_ nanoparticles, two types of measurement were carried out, namely, using zero-field-cooled (ZFC) and field-cooled (FC) measurements with a rate of 2 K/min. For the ZFC measurement, the sample was initially cooled down from 300 K down to 2 K in a zero magnetic field, and the magnetization was recorded while warming the system in a magnetic field of 100 Oe. For the FC measurement, the sample was again cooled down from 300 K down to 2 K, but in a magnetic field of 100 Oe, magnetization was recorded while warming the system in the same applied magnetic field. Figure 8(a)–(d) shows the temperature dependency of magnetization M(*T*) with ZFC-FC modes for 15 ± 1, 54 ± 4, 62 ± 3, 85 ± 6 nm Co_3_O_4_ NPs in an applied field of 100 Oe. A high irreversible temperature (*T*_*irr*_) defined by the temperature at which the FC curve starts to split from the ZFC curve, above 200 K is observed for 15 ± 1, 62 ± 3 and 85 ± 6 nm NPs, whereas 54 ± 4 nm NPs show *T*_*irr*_ ~ 60 K, relating to spin-glass-like behavior. Furthermore, from the M(*T*) curve for 15 ± 3 nm sample, two common characteristic features can be drawn. First, a blocking temperature *T*_*B*_ is defined as the maximum value of ZFC curve, which increases from 31 ± 1 to 38 ± 1 K as the particle size increases from 15 ± 1 to 85 ± 6 nm. Second, there is a sharp increasing behavior of magnetization at low temperature, defined as freezing temperature *T*_*f*_ = 11 K, as shown in Fig. 8(a). Similar temperature dependent magnetization behavior has been reported in several experimental ZFC-FC curves from Co_3_O_4_ nanoparticles^58,59^. However, there are also reports in which anomalous magnetic properties such as weak-ferromagnetism and SPM behavior have been reported from Co_3_O_4_ nanoparticles^60^. According to the Néel-Brown model^61,62^, the uniaxial and non-interacting superparamagnetic nanoparticles exhibit a distribution of the anisotropy energy barrier due to polydispersity. If each particle gets blocked at the blocking temperature, *T*_*B*_ then in poly-dispersed nanoparticles, ${T}_{B}\approx KV/25{k}_{B}$ will be associated with the mean value of *T*_*B*_, where *K* is the effective anisotropy energy density, *V* is particle volume and *k*_*B*_ is Boltzmann’s constant. The effective anisotropy comprises several intrinsic factors such as volume, surface, shape, exchange and magnetocrystalline anisotropies. According to the above expression, the value of *T*_*B*_ increases with particle size, which is in good agreement with the observed increasing behavior of *T*_*B*_ = 34 ± 1, 36 ± 1 and 38 ± 1 K from Co_3_O_4_ nanoparticles of sizes 54 ± 4, 62 ± 3 and 85 ± 6 nm, respectively. The calculated value of *K*, summarized in the supplementary information of Table S4, decreases from 5331 J/m^3^ to 80 J/m^3^ with the increase of particle size from 15 ± 1 nm to 85 ± 6 nm, respectively. The *K* is much smaller than reported value of 90 kJ/m^3^ from the 3 nm sized Co_3_O_4_ nanoparticles (*T*_*B*_ = 3 K)^63^. It shows the opposite behavior compared to the blocking temperature due to a reduction of particle mean volumes at lower sizes. This anomalous enhancement of the effective anisotropy indicates that the maximum in the ZFC magnetization does not correspond to the typical blocking temperature of the non-interacting particles, where inter particle interaction, including dipole-dipole and exchange interaction from weak-ferromagnetic shell moment of Co_3_O_4_ nanoparticles, plays a significant role in the temperature dependence of the magnetization. The irreversible temperature, *T*_*irr*_ determines the highest value for *T*_*B*_ in the system, whereas *T*_*f*_ at lower temperature corresponds to the surface spin freezing temperature. *T*he temperature *T*_*f*_  ~ 10 K is associated with the freezing of uncompensated surface spins which has been reported a number of times from different size Co_3_O_4_ nanoparticles.

### Finite size effect on the Néel temperature with Co_3_O_4_ nanoparticles

**Figure 9:**
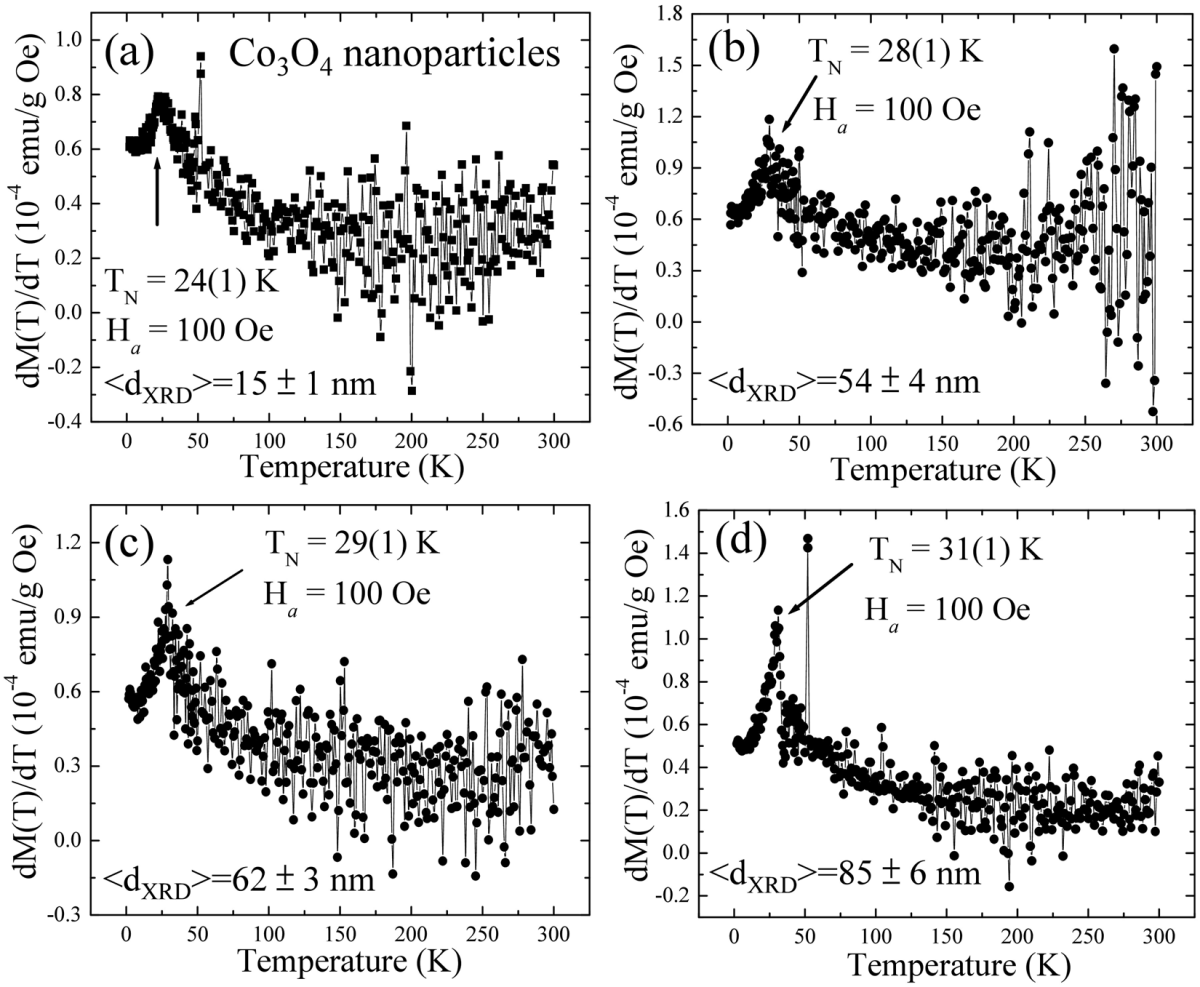
(**a**)–(**d**) Temperature dependence of differential magnetization $\partial {\rm{M}}({\rm{T}})/\partial {\rm{T}}$ for 15 ± 1, 54 ± 4, 62 ± 3 and 85 ± 6 nm Co_3_O_4_ NPs.

**Figure 10:**
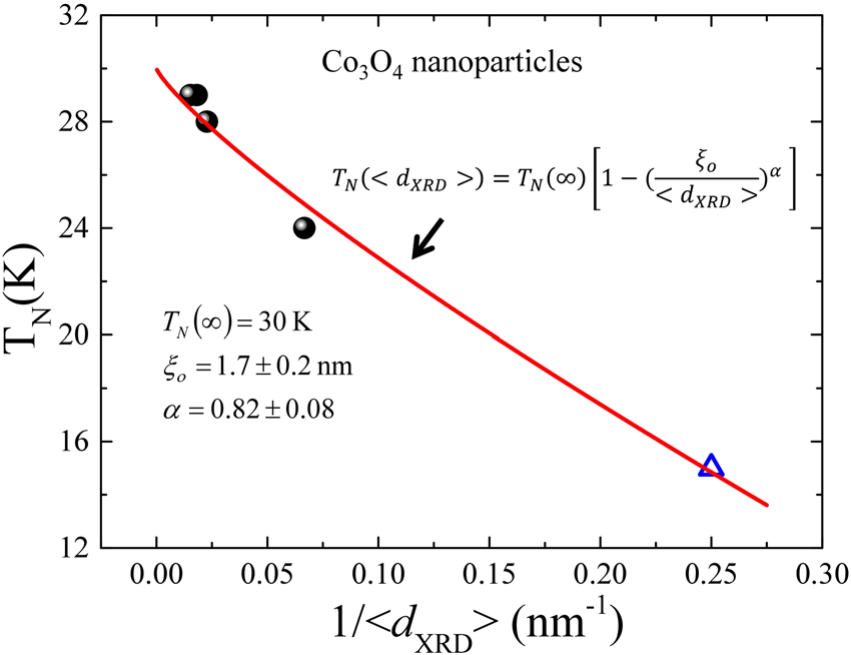
Plot of Néel transition temperature *T*_*N*_ verses inverse of particles size of Co_3_O_4_ NPs, where the solid line is fit by using the finite size scaling relation. The open triangle are the data points taken from reference number 6.

In magnetic nanomaterials, by reducing the particle size, an intrinsic finite size effect causing a reduction in the magnetic ordering temperature occurs. The Co^3+^ ions at the octahedral sites are diamagnetic while the spin moments of the Co^2+^ ions at the tetrahedral sites exhibit AFM ordering at *T* < *T*_*N*_ = 40 K^17^. In the nanoscale region, some experiments have reported a reduced *T*_*N*_. For example, *T*_*N*_ ~ 30 K has been observed with 8 nm Co_3_O_4_ nanoparticles and 15 ± 2 K with 4.3 nm nanoparticles^18–20^. Experimentally, the value of Néel temperature *T*_*N*_ can be obtained as the peak in the $\partial {\rm{M}}({\rm{T}})/\partial {\rm{T}}$ versus *T* plot^64^, as shown in Fig. 9(a)–(d). These plots yield *T*_*N*_ = 24 ± 1, 28 ± 1, 29 ± 1 and 31 ± 1 K from Co_3_O_4_ nanoparticles of size 15 ± 1, 54 ± 4, 65 ± 3 and 85 ± 6 nm, respectively. The value of *T*_*N*_ for 65 ± 3 and 85 ± 6 nm is close to the reported value of 30 K for bulk Co_3_O_4_^18,19^, which is in good agreement with structural and atomic percent results as observed from SR-XRD and EDS measurements. The decrease of *T*_*N*_ = 24 ± 1 K for 15 ± 1 nm Co_3_O_4_ nanoparticles can be understood by considering the intrinsic finite size effect on the correlation length of the magnetic ordering temperature, which has been reported from FM, AFM^53,58^ and ferromagnetic^65^ nanostructured materials. Figure 10 displays the inverse of particles size dependency of *T*_*N*_, where the solid line is fitted to the finite size scaling relation defined as: ${T}_{N}( < {d}_{XRD} > )={T}_{N}(\infty )[1-{(\frac{{\xi }_{0}}{ < {d}_{XRD} > })}^{\alpha }],\,{\rm{where}}\,{T}_{N}(\infty )=30\,{\rm{K}}$ for bulk Co_3_O_4_, ${\xi }_{o}$ is the correlation length of bulk phase at zero temperature and α the shift exponent. Fitting yields α = 0.82 ± 0.08 and ${\xi }_{o}=1.7\pm 0.2\,nm$. The value of α is close to the theoretical predicted value of 1 by mean field theory^66^, but lower than the 3D Heisenberg model^67^ (1.4) and Ising Hamiltonian^68^ (1.6) values. The correlation length ${\xi }_{o}$ is about ~2 times the lattice constant of Co_3_O_4_, which is agreed with the predicted value of 1.6 nm, derived from Monte Carlo simulation^69^.

## Discussion

The crystalline, optical and magnetic properties of microwave assisted synthesized Co_3_O_4_ NPs (15 to 85 nm) have been systematically investigated. The observed non-stoichiometry nature from EDS spectra and expansion in (Co^2+^-O^2−^) bond length from SR-XRD confirm that weak magnetism from 15 ± 1 nm NPs (at 2 K) is associated with the cobalt vacancies Co^2+^ at the tetrahedral site. Nanoscale size effects lead to lattice expansion, line-broadening, red shift in the phonon wave number and reduction in the Néel temperature *T*_*N*_ of Co_3_O_4_ NPs, giving a short-range magnetic correlation length ${\xi }_{o}$ ~ 1.7 nm from the fitting of the finite size scaling model. The size dependency of *T*_*N*_ and the observed line-broadening and red shift in the phonon wave number show the effect of the finite size effect^10^. However, it remains questionable, why only 15 ± 1 nm Co_3_O_4_ NPs shows a hysteresis loop below the Neel transition temperature. In general, based on the experimental conditions, the dynamically post-annealing of microwave assisted grown nanocrystals could involve many competing atomic movements, including the transport of atomic-size matter from core to surface and on-surface reconstruction. In this study, the annealing time is fixed, thus *T*_*A*_ is the only factor to affect the size and points of defects (cobalt vacancies) in the nanocrystals. By taking into account the observed experimental results from SR-XRD and the EDS spectra, we propose that cobalt vacancies in Co_3_O_4_ NPs are located at the tetrahedral Co^2+^ site. These vacancies in the 15 nm sample are mostly distributed on the surface since the surface-to-volume ratio is high, thus weak magnetism comes from defect interactions. The above finding also opens an avenue for further increase of intrinsic FM properties by controlling the density of defects under experimental conditions. Moreover, the mechanism of the AFM transition of the Co-3*d* electrons in Co_3_O_4_ is discussed, and we show that the occurrence of finite size effect on the magnetic ordering. The reduction of *T*_*N*_ with the decrease of particles size is attributed to the intrinsic finite size effect. These results open a new route to achieve and manipulate short-range magnetic states in spintronic devices.

## Method

Cobalt (II) nitrate hexahydrate (Co(NO_3_)_2_∙6H_2_O, 99.99%), Poly Ethyl Glycol [PEG] and ammonia solution (30%) [NH_4_OH] were the only chemicals used during the synthesis. All the chemicals were purchased from Sigma-Aldrich and used as received. Deionized water (DIW) is used as a solvent. Co_3_O_4_ NPs were synthesized using the microwave irradiation technique. Cobalt nitrate hexahydrate and ammonia solution were used as precursors and distilled water as a solvent. Initially, a 0.4 M aqueous solution of cobalt nitrate under continuous stirring was prepared at room temperature. Ammonia solution was added drop by drop until the pH of the solution became 10. The solution turns into a dark green slurry, suggesting the formation of a stable cobalt ammonia complex. For the stabilization of Co_3_O4 nanoparticles, 0.5 g of PEG was dissolved in 10 mL of distilled water in 100 mL of beaker; then 3 mL of PEG solution was added to the slurry. This mixture was exposed to microwave irradiation at 80 °C for 15 minutes at 140 watt power under constant stirring. Due to microwave heating, the cobalt ammonia complex decomposes forming a dark green precipitate. This precipitate was separated from the supernatant liquid by centrifuging at 2000 rpm for 2 minutes. The residue was washed with distilled water and then with ethanol 2-3 times. The dark green color residue was then dried overnight in air. And the obtained resultant sample, namely as-grown Co_3_O_4_ sample, was divided into four sets and annealed using a high-temperature horizontal quartz tube furnace. The complete post-synthesis process is as follows:(1) The as-grown sample was put on a porcelain boat and placed in a quartz tube in the middle region of a heated oven. (2) The pressure of the quartz tube was reduced to less than 1 × 10^−2^ Torr by a mechanical pump. (3) The heating temperature in a quartz tube was set for various as-grown samples in a temperature range of T_A_ = 450–800 °C, respectively. (4) After the temperature was stabilized, a mixed gas of oxygen (20 *sccm*) and argon (80 *sccm*) was introduced into the tube, and the pressure was kept at 760 Torr by a flux controller. (5) The boat was heated at a set temperature for two hours with a heating rate of 5 K/min and cooled down to room temperature naturally after the heating. (6) Finally, these resultant samples were saved in a low-pressure container to avoid further oxidation. Field-emission scanning electron microscopy (FE-SEM, JEOL JSM-6500F) microscope and energy dispersive spectroscopy (EDS; Inca x-sight model 7557, Oxford Instruments, Abingdon, Oxfordshire, U.K.) were utilized to investigate the morphology, size distribution of nanoparticles, and the atomic percentages of cobalt and oxygen. Detailed crystal structure measurement was carried out through a synchrotron radiation x-ray diffraction (SR-XRD) with a BL01C2 beam line and an incident wavelength of λ = 0.7749 Å at the National Synchrotron Radiation Research Center in Taiwan (for more detail see the ref.^21^). Confocal Raman spectrometer (α−300, WiTec Pte. Ltd., Ulm, Germany) with a 488-nm Ar ion laser (CVI Melles Griot, Carlsbad, CA)was used to measure the multi-phonon properties of Co_3_O_4_ nanoparticles.

**Note:** SRG and ACG share equal weightage in this manuscript. ACG is currently affiliated to the Center for Condensed Matter Sciences, National Taiwan University, Taipei, Taiwan.

## Electronic supplementary material

Supplementary information
