# Supplementary material for: Role of cobalt cations in short range antiferromagnetic Co3O4 nanoparticles: a thermal treatment approach to affecting phonon and magnetic properties
Source: Sci Rep. 2018 Jan 10;8:249. doi: 10.1038/s41598-017-18563-9 (PMC5762665; doi:10.1038/s41598-017-18563-9)
Supplement: Supplementary file 1 — Supplementary information [file 41598_2017_18563_MOESM1_ESM.pdf]

Supplementary information

Role of cobalt cations in short range antiferromagnetic Co_3O_4 nanoparticles: a thermal treatment approach to affecting phonon and magnetic properties

Swati R. Gawali[1], Ashish Chhaganlal Gandhi[2], Shrikrushna Shivaji Gaikwad[2], Jayashree Pant[3], Ting-Shan Chan[4], Chia-Liang Cheng[2], Yuan-Ron Ma[2] & Sheng Yun Wu[*,2]

[1]Department of Physics, CES's Dr. A. B. Telang Sr. College, Savitribai Phule Pune University, Pune 411007, India

[2]Department of Physics, National Dong Hwa University, Hualien 97401, Taiwan

[3]Department of Physics, Abasaheb Garware College, Savitribai Phule Pune University, Pune 411007, India

[4]National Synchrotron Radiation Research Center, Hsinchu 30076, Taiwan

Table S1 Summary of the fitting parameters obtained after the Lorentzian fit of (311) SR-XRD peak, crystalline size from W-H plot, and Rietveld refined parameters.

Co_3O_4 : Space group Fd3m (No. 227)									
			Lattice constant	GSAS fitting parameters			Atomic Position (x=y=z)		
T_A (°C)	$<d_{XRD}>$(nm)	*fwhm(331)*	*a=b=c (Å)*	wRp	Rp	χ^2	Co^{2+} 8(a)	Co^{3+} 16(d)	O^{2-} 32(e)
450	15 ± 1	0.308(2)	8.1022(5)	0.0428	0.0327	1.308	1/8	1/2	0.2614(3)
600	54 ± 4	0.109(3)	8.1018(1)	0.0366	0.0239	0.9846	1/8	1/2	0.2622(2)
700	62 ± 3	0.079(3)	8.1007(1)	0.0576	0.0476	2.988	1/8	1/2	0.2623(3)
800	85 ± 6	0.078(3)	8.1007(1)	0.0544	0.0320	2.758	1/8	1/2	0.2622(2)

Table S2 Summary of the fitting parameters obtained from the Rietveld refinement of XRD patterns and bond valence method.

T_A (°C)	Lattice constant<br>a=b=c (Å)	Co^{2+}-O^{2-} Length (Å)<br>d_{jk}	Valence<br>Co^{2+} (0) (v.u.)	Co^{3+}-O^{2-} Length (Å)<br>d_{jk}	Valence<br>Co^{3+} (0) (v.u.)	Electron density (e^-/$Å^3$)
Bulk	8.0900(1)	1.929		1.916	0	----
450	8.1022(5)	1.9178	0.12338	1.9354	-0.30634	447.742
600	8.1018(1)	1.9125	1.82416	1.938	-0.34636	257.328
700	8.1007(1)	1.9127	0.18016	1.9389	-0.36009	546.134
800	8.1007(1)	1.9106	0.20395	1.9389	-0.36009	380.026

Table S3 Summary of the fitted values of the Raman shift (center) and FWHM after de-convoluting the Raman spectra by using the Voigt function.

T_A(°C)	<d_{XRD}> nm	F_{2g}^3(cm^{-1})		E_g(cm^{-1})		F_{2g}^1 (cm^{-1})		F_{2g}^2 (cm^{-1})		A_{1g}(cm^{-1})	
		Center	*fwhm*	Center	*fwhm*	Center	*fwhm*	Center	*fwhm*	Center	*fwhm*
450	15 ± 1	188.8(8)	18.4	469(0.3)	20.0	511.5(4)	22.7	605(1)	17.7	673.9(1)	21.2
600	54 ± 4	192.8(8)	10.9	476.8(3)	14.7	517.3(3)	15.4	614(1)	16.2	684.5(1)	14.4
700	62 ± 3	194.4(9)	11.7	480.4(1)	11.7	519.8(2)	14.0	618.3(6)	11.8	688.72(4)	11.9
800	85 ± 6	192(3)	3.2	482.4(3)	12.3	521.6(3)	8.3	618(2)	7.3	691.8(1)	11.7

Table S4 Summary of the obtained values from the temperature dependency of magnetization M(*T*) with ZFC-FC modes for 15±1, 54±4, 62±3, 85±6 nm Co_3O_4 NPs in an applied field of 100 Oe.

T_A(°C)	<d_{XRD}>(nm)	T_f(K)	T_N(K)	T_B(K)	K(J/m^3)	T_{irr}(K)
450	15 ± 1	10 ± 1	24 ± 1	31 ± 1	5331	75
600	54 ± 4	10 ± 1	28 ± 1	34 ± 1	232	
700	62 ± 3	10 ± 1	29 ± 1	36 ± 1	126	
800	85 ± 6	10 ± 1	31 ± 1	38 ± 1	80	
Bulk			40			
